# Supplementary material for: Selective concentration of iron, titanium, and zirconium substrate minerals within Gregory’s diverticulum, an organ unique to derived sand dollars (Echinoidea: Scutelliformes)
Source: PeerJ. 2024 Apr 5;12:e17178. doi: 10.7717/peerj.17178 (PMC11000648; doi:10.7717/peerj.17178)
Supplement: Supplemental Information 3 — List of access links to MorphoBank repository for µC. datasets [file peerj-12-17178-s003.docx]

| **Species** | **Specimen** | **Test length** | **MorphoBank download link** |
| --- | --- | --- | --- |
| *Sinaechinocyamus mai* | CASIZ 188797 | 6.5 mm | <https://morphobank.org/index.php/Projects/Media/DownloadMedia/id/895004/project_id/4915/tablename/media_files/download/1> |
| *Sinaechinocyamus mai* | NMNS 2689-178 | 7.5 mm | <https://morphobank.org/index.php/Projects/Media/DownloadMedia/id/895008/project_id/4915/tablename/media_files/download/1> |
| *Scaphechinus mirabilis* | ZMB Ech 7405 | 21 mm | <https://morphobank.org/index.php/Projects/Media/DownloadMedia/id/895000/project_id/4915/tablename/media_files/download/1> |
| *Echinarachnius parma* | Unvouchered | 4 mm | <https://morphobank.org/index.php/Projects/Media/DownloadMedia/id/894907/project_id/4915/tablename/media_files/download/1> |
| *Echinarachnius parma* | MCZ Ech-2613 | 10 mm | <https://morphobank.org/index.php/Projects/Media/DownloadMedia/id/894903/project_id/4915/tablename/media_files/download/1> |
| *Dendraster excentricus* | CASIZ 094162 | 6 mm | <https://morphobank.org/index.php/Projects/Media/DownloadMedia/id/894895/project_id/4915/tablename/media_files/download/1> |
| *Dendraster excentricus* | CASIZ 094162 | 9 mm | <https://morphobank.org/index.php/Projects/Media/DownloadMedia/id/894899/project_id/4915/tablename/media_files/download/1> |
| *Encope michelini* | TNSC NPL-4110 | 9 mm | <https://morphobank.org/index.php/Projects/Media/DownloadMedia/id/894911/project_id/4915/tablename/media_files/download/1> |
| *Encope michelini* | TNSC NPL-4111 | 9 mm | <https://morphobank.org/index.php/Projects/Media/DownloadMedia/id/894916/project_id/4915/tablename/media_files/download/1> |
| *Encope michelini* | TNSC NPL-4112 | 13 mm | <https://morphobank.org/index.php/Projects/Media/DownloadMedia/id/894920/project_id/4915/tablename/media_files/download/1> |
| *Encope michelini* | Unvouchered | 14 mm | <https://morphobank.org/index.php/Projects/Media/DownloadMedia/id/894928/project_id/4915/tablename/media_files/download/1> |
| *Encope michelini* | TNSC NPL-4113 | 16 mm | <https://morphobank.org/index.php/Projects/Media/DownloadMedia/id/894924/project_id/4915/tablename/media_files/download/1> |
| *Encope micropora* | MCZ Ech-2625 | 36 mm | <https://morphobank.org/index.php/Projects/Media/DownloadMedia/id/894932/project_id/4915/tablename/media_files/download/1> |
| *Lanthonia grantii* | USNM E47210 | 16 mm | <https://morphobank.org/index.php/Projects/Media/DownloadMedia/id/894936/project_id/4915/tablename/media_files/download/1> |
| *Leodia sexiesperforata* | CASIZ 112813 | 5 mm | <https://morphobank.org/index.php/Projects/Media/DownloadMedia/id/894960/project_id/4915/tablename/media_files/download/1> |
| *Leodia sexiesperforata* | ZMH E6707 | 8 mm | <https://morphobank.org/index.php/Projects/Media/DownloadMedia/id/894964/project_id/4915/tablename/media_files/download/1> |
| *Mellita notabilis* | Unvouchered | 32 mm | <https://morphobank.org/index.php/Projects/Media/DownloadMedia/id/894968/project_id/4915/tablename/media_files/download/1> |
| *Mellita quinquiesperforata* | Unvouchered | 2 mm | <https://morphobank.org/index.php/Projects/Media/DownloadMedia/id/894972/project_id/4915/tablename/media_files/download/1> |
| *Mellita tenuis* | CASIZ | 6 mm | <https://morphobank.org/index.php/Projects/Media/DownloadMedia/id/894976/project_id/4915/tablename/media_files/download/1> |
| *Mellita tenuis* | MCZ Ech-8000 | 11 mm | <https://morphobank.org/index.php/Projects/Media/DownloadMedia/id/894988/project_id/4915/tablename/media_files/download/1> |
| *Mellita tenuis* | Unvouchered | 21 mm | <https://morphobank.org/index.php/Projects/Media/DownloadMedia/id/894992/project_id/4915/tablename/media_files/download/1> |
| *Mellitella stokesii* | USNM E40733 | 17 mm | <https://morphobank.org/index.php/Projects/Media/DownloadMedia/id/894996/project_id/4915/tablename/media_files/download/1> |
