# Supplementary figures and images for: Selective concentration of iron, titanium, and zirconium substrate minerals within Gregory’s diverticulum, an organ unique to derived sand dollars (Echinoidea: Scutelliformes)
Source: PeerJ. 2024 Apr 5;12:e17178. doi: 10.7717/peerj.17178 (PMC11000648; doi:10.7717/peerj.17178)

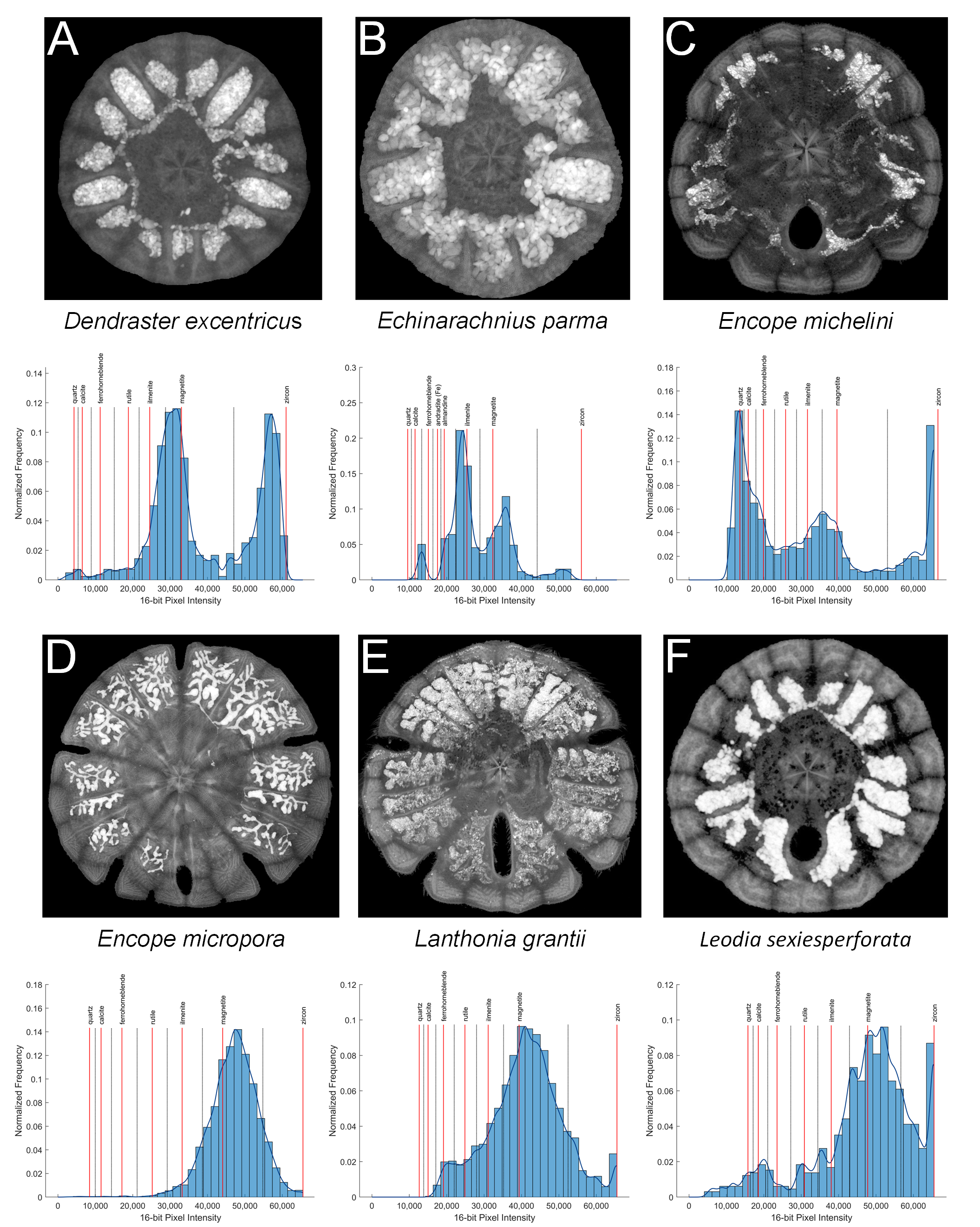

Supplement: Supplemental Information 4 — (A) Dendraster excentricus, (B) Echinarachnius parma, (C) Encope michelini, (D) Encope micropora, (E) Lanthonia grantii, (F) Leodia sexiesperforata. [file peerj-12-17178-s004.png]

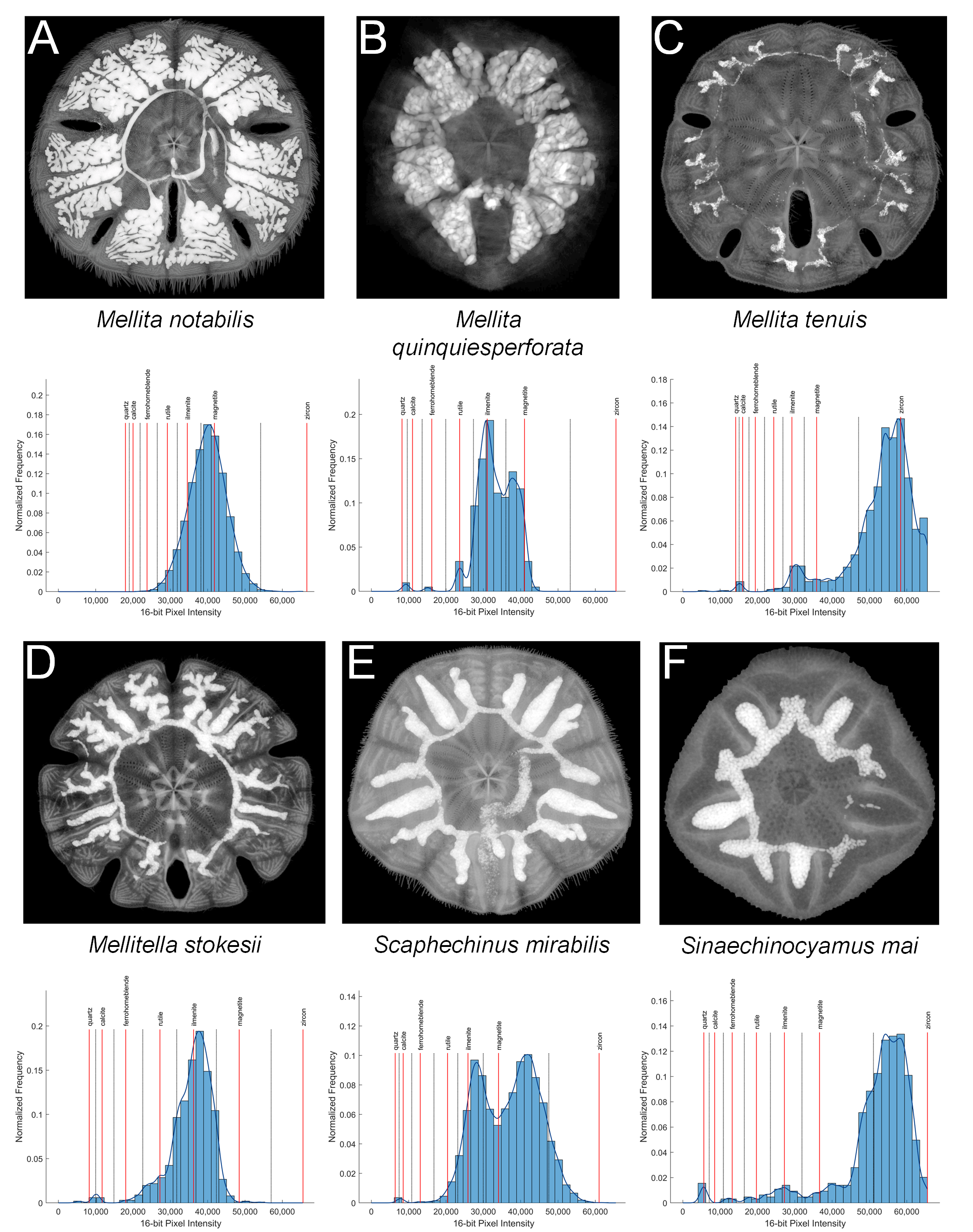

Supplement: Supplemental Information 5 — (A) Mellita notabilis, (B) Mellita quinquiesperforata, (C) Mellita tenuis, (D) Mellitella stokesii, (E) Scaphechinus mirabilis, (F) Sinaechinocyamus mai. [file peerj-12-17178-s005.png]
